# Copper(II) Thiosemicarbazone Complexes and Their Proligands upon UVA Irradiation: An EPR and Spectrophotometric Steady-State Study

**DOI:** 10.3390/molecules23040721

**Published:** 2018-03-21

**Authors:** Michal Hricovíni, Milan Mazúr, Angela Sîrbu, Oleg Palamarciuc, Vladimir B. Arion, Vlasta Brezová

**Affiliations:** 1 Institute of Physical Chemistry and Chemical Physics, Faculty of Chemical and Food Technology, Slovak University of Technology in Bratislava, Radlinského 9, SK-812 37 Bratislava, Slovak Republic; michal.hricovini@stuba.sk (M.H.); milan.mazur@stuba.sk (M.M.); 2 Department of Chemistry, Moldova State University, A. Mateevici Street 60, MD-2009 Chisinau, Moldova; sirbuangela@yandex.ru (A.S.); palamarciuco@gmail.com (O.P.); 3 Institute of Inorganic Chemistry, University of Vienna, Währinger Strasse 42, A-1090 Vienna, Austria; vladimir.arion@univie.ac.at

**Keywords:** sodium 5-sulfonate-salicylaldehyde thiosemicarbazones, copper(II) complexes, Q- and X-band EPR spectroscopy, spin trapping, UVA exposure, reactive oxygen species

## Abstract

X- and Q-band electron paramagnetic resonance (EPR) spectroscopy was used to characterize polycrystalline Cu(II) complexes that contained sodium 5-sulfonate salicylaldehyde thiosemicarbazones possessing a hydrogen, methyl, ethyl, or phenyl substituent at the terminal nitrogen. The ability of thiosemicarbazone proligands to generate superoxide radical anions and hydroxyl radicals upon their exposure to UVA irradiation in aerated aqueous solutions was evidenced by the EPR spin trapping technique. The UVA irradiation of proligands in neutral or alkaline solutions and dimethylsulfoxide (DMSO) caused a significant decrease in the absorption bands of aldimine and phenolic chromophores. Mixing of proligand solutions with the equimolar amount of copper(II) ions resulted in the formation of 1:1 Cu(II)-to-ligand complex, with the EPR and UV-Vis spectra fully compatible with those obtained for the dissolved Cu(II) thiosemicarbazone complexes. The formation of the complexes fully inhibited the photoinduced generation of reactive oxygen species, and only subtle changes were found in the electronic absorption spectra of the complexes in aqueous and DMSO solutions upon UVA steady-state irradiation. The dark redox activity of copper(II) complexes and proligand/Cu(II) aqueous solutions towards hydrogen peroxide which resulted in the generation of hydroxyl radicals, was confirmed by spin trapping experiments.

## 1. Introduction

Aromatic and heteroaromatic thiosemicarbazones (TSCs) and their transition metal complexes have gained considerable attention due to their coordination chemistry and broad range of biological activities [1,2,3,4,5,6,7,8,9]. The biological actions of thiosemicarbazones are often related to the chelation of metal ions, especially regarding coordination with biologically relevant transition metal ions [10,11,12,13,14,15]. For example, Cu(II)-TSC complexes are effective anticancer agents, and act as strong inhibitors of DNA synthesis via the inhibition of ribonucleotide reductase [16,17,18]. The position and type of substituent on the TSC backbone, along with the character of the metal ion, have a strong impact on the overall properties and stability of the coordination compound, as well as its biological activity. The presence of amide, imine, and thione groups in the thiosemicarbazone molecule are responsible for its excellent metal ion chelating capacity [19,20,21].

**Scheme 1:**
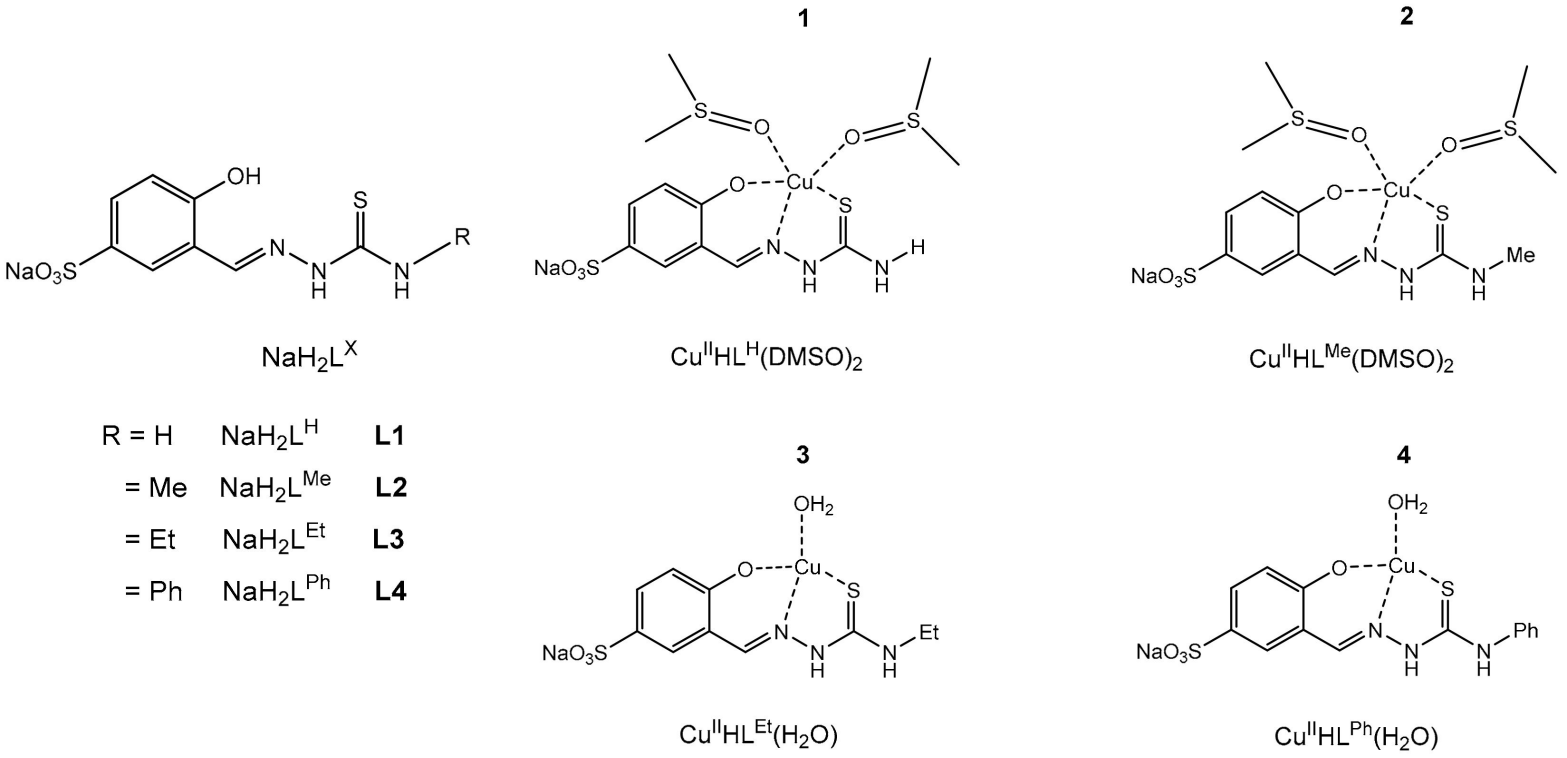
Line drawings of the investigated thiosemicarbazone proligands **L1**–**L4** and their Cu(II) complexes (**1**–**4**).

Despite the low water-solubility of (hetero)/aromatic TSCs, suitable modification of their structure may result in enhanced solubility in aqueous media, which is highly desirable for applications in vivo. For example, when salicylaldehyde thiosemicarbazone is coupled with prolines a significant increase in water solubility is observed [22,23,24]. In particular, when coordinated to Cu(II) ions, these TSC-derivatives exhibit cytotoxic activity against ovarian carcinoma CH1 cells [25,26,27]. A new series of sodium 5-sulfonate-salicylaldehyde thiosemicarbazones containing different substituents at the terminal nitrogen atom (**L1**–**L4** in Scheme 1) was synthesized recently, with the aim to obtain water-soluble TSC derivatives with potential biological activity. Proligands **L1**–**L4** and their Cu(II) complexes (**1**–**4** in Scheme 1) were characterized by different spectroscopic techniques, electrospray ionization (ESI) mass spectrometry, X-ray crystallography and cyclic voltammetry [28]. The behavior of proligands **L1**–**L4** in aqueous media is determined by the presence of two dissociable hydrogens; the first dissociation step was assigned to the deprotonation of the phenolic group with p*K*_1_ values in the range of 7.73–7.82, without any noticeable effect of substituents at the terminal nitrogen [28]. The second deprotonation step, attributed to the loss of a proton from the hydrazinic NH-group of thiosemicarbazide moiety, is observable in a strongly basic medium [28]. While TSC-derivatives **L1**–**L4** demonstrated only limited cytotoxicity against the human cancer cell lines, the corresponding Cu(II) complexes showed moderate anticancer activity, coupled with the ability to induce the accumulation of reaction oxygen species (ROS) in target cells, which was confirmed by using 2′,7′-dichlorodihydrofluorescein diacetate, which, in the presence of ROS, was subsequently converted into the highly fluorescent 2′,7′-dichlorofluorescein [28].

Development of anticancer drugs is a multiparameter task, which requires, among other things, elucidation of the photostability of the potential drugs in a biocompatible media, in order to avoid the undesired rapid decomposition of the active species. The photochemical instability of some pharmaceutical products may lead to undesired side effects during their applications, as well as problems in handling and administration [29,30,31,32,33]. Herein, we studied the effect of UVA exposure on both metal-free proligands, and their copper(II) complexes, and, in particular, the ability of compounds to generate ROS upon irradiation. The electron paramagnetic resonance (EPR) spin trapping technique with 5,5-dimethyl-1-pyrroline *N*-oxide (DMPO) as the spin trap was applied to follow the generation of ROS upon UVA irradiation of complexes **1**–**4,** and proligands **L1**–**L4** in water or DMSO. Cu(II) complexes revealed high photostability and limited capacity to produce ROS; however, upon UVA exposure, photoexcitation of proligands **L1**–**L4** caused significant changes in their electronic spectra; these changes were attributable to interactions with generated ROS (hydroxyl radicals, superoxide radical anions) found using spin trapping. In addition, hydroxyl radical formation was confirmed in cell-free experiments investigating the dark redox activity of Cu(II) complexes with hydrogen peroxide by undertaking EPR spin trapping experiments. This agrees well with the previously reported data on cancer cells [28]. 

## 2. Results and Discussion

### 2.1. EPR Spectra of Polycrystalline Cu(II) Complexes

**Figure 1:**
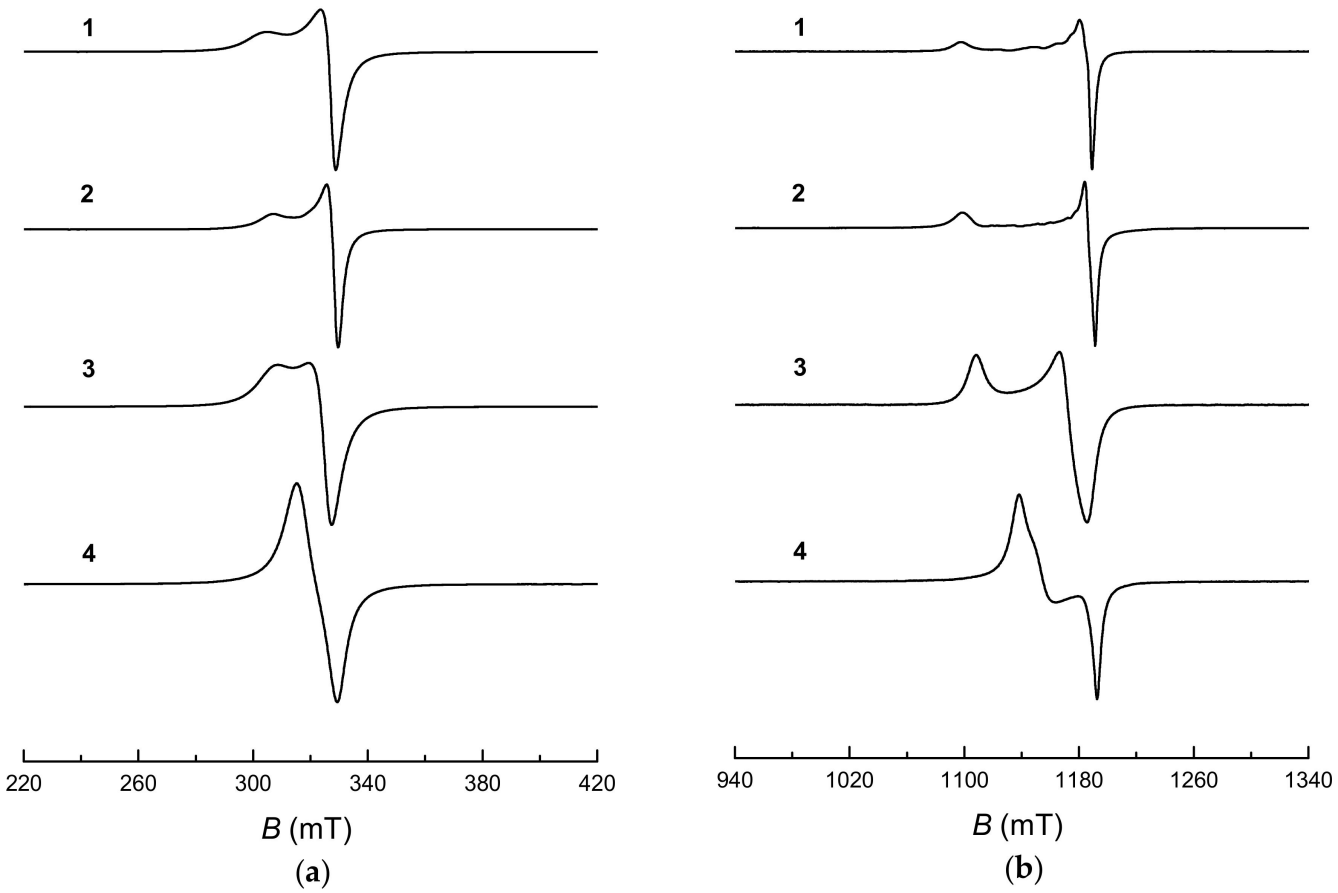
X-band (**a**) and Q-band (**b**) electron paramagnetic resonance (EPR) spectra of polycrystalline Cu(II) complexes **1**–**4** measured at 100 K. EPR spectrometer settings: (**a**) microwave frequency, 9.458 GHz; microwave power, 10.03 mW; center field, 320 mT; gain, 7.1 × 10^2^; modulation amplitude, 0.5 mT; scan, 84 s; time constant, 40.96 ms; (**b**) microwave frequency, 33.999 GHz; microwave power, 2.28 mW; center field, 1140 mT; gain, 2.0 × 10^1^; modulation amplitude, 0.05 mT; scan, 121 s; time constant, 163.84 ms. The spin-Hamiltonian parameters evaluated from the simulations are summarized in Table 1.

Figure 1 summarizes the X- and Q-band electron paramagnetic resonance (EPR) spectra of polycrystalline Cu(II) complexes **1**–**4** measured at 100 K, although fully compatible EPR spectra were obtained already at room temperature. For complexes **1**–**3**, axially symmetric EPR spectra with unresolved hyperfine couplings were measured in both frequency domains, while better *g*-tensor components resolution was provided by the Q-band (Figure 1b). The values of *g*_‖_ and *g*_⊥_, refined by computer simulations, of the experimental EPR spectra of complexes **1**–**3** (Table 1) matched well with the relation *g*_‖_ > *g*_⊥_ > 2.0023, which is consistent with the d_x2−y2_ ground electronic state of the Cu(II) ion. To obtain more detailed information, we evaluated the parameter G = (*g*_‖_ − 2)/(*g*_⊥_ − 2) as a measure of the exchange interaction between copper centers in the polycrystalline solid [34,35]. The G-values of 2.91 or 3.31, both below 4, were calculated from X- and Q-band spectra of complex **3**, respectively, indicating an exchange interaction in this complex in the solid state. In contrast, G-values higher than 4 were obtained from the spectra of complexes **1** and **2;** this indicated only a negligible exchange interaction, which correlated well with the differences in structure found previously by the single crystal X-ray diffraction of complexes **1**–**3** [28]. 

Solid-state structural analysis of **1** and **2** confirmed the coordination of copper(II), provided by the ONS-donor set of the Schiff base ligand and two molecules of DMSO bound in equatorial and apical positions, with the geometry of the central atom corresponding to a slightly-distorted square-pyramidal coordination [28]. The crystal structures of **1** and **2** are stabilized by a system of N‒H∙∙∙O hydrogen bonds forming a 3D supramolecular network. On the other hand, the structure of **3** consists of two asymmetric Cu(II) entities, with the square-pyramidal coordination geometry of the central atom linked through the centre of symmetry, forming a tetranuclear complex. The main crystal structural motif for **3** can be described as a 3D supramolecular network based on the O‒H···O and O‒H···N hydrogen bonding [28]. The X- and Q-band EPR spectra of polycrystalline complex **4** revealed rhombic features that were well resolved, especially at higher frequency; no hyperfine coupling was found (Figure 1b). The orthorhombic *g*-tensor components that were evaluated from the simulation of the experimental spectra are gathered in Table 1.

**Figure 2:**
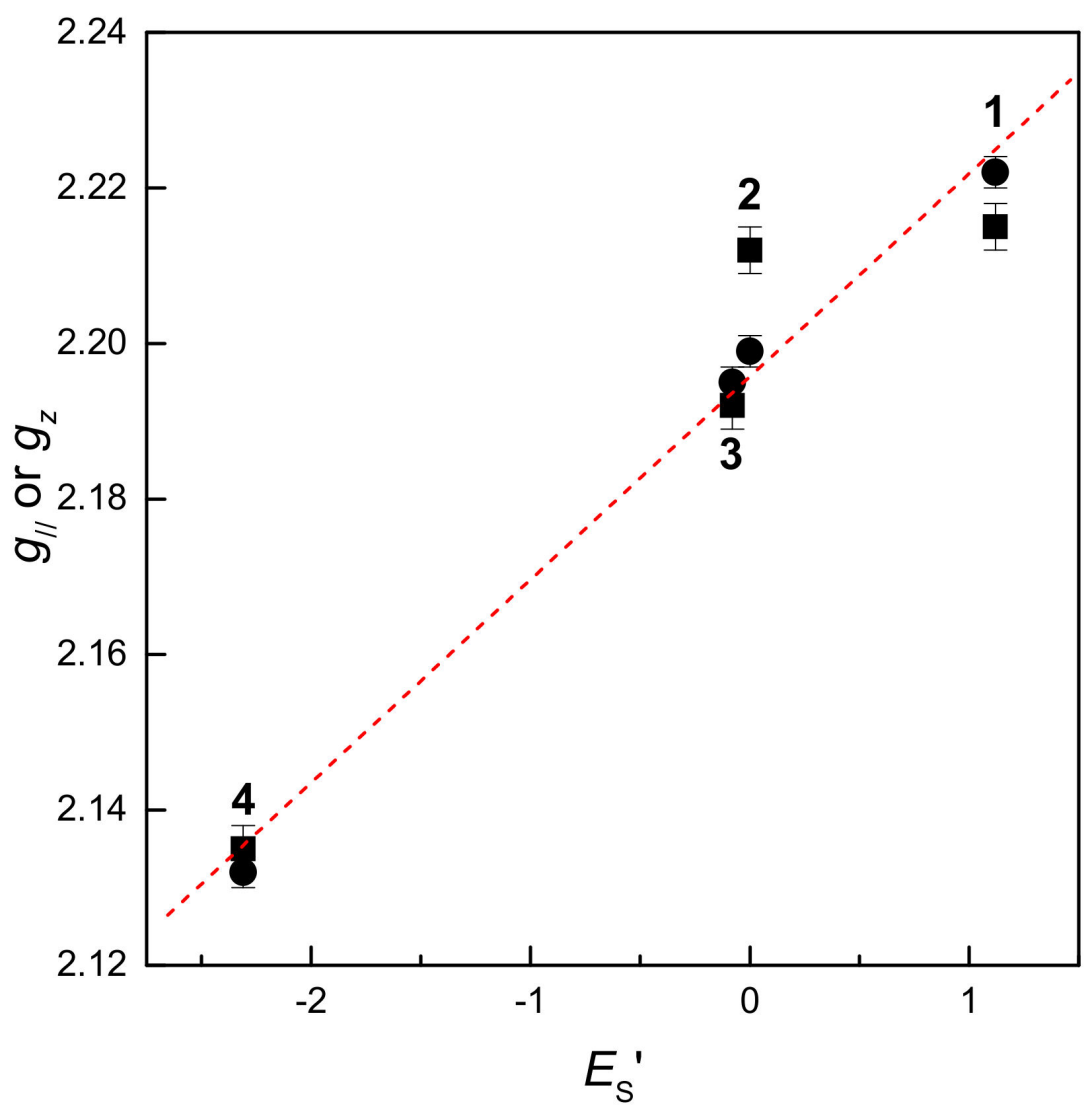
Relationship of *g*_‖_ or *g*_z_ values evaluated from simulation of X-band (●) and Q-band (■) EPR spectra of polycrystalline Cu(II) complexes **1**–**4** vs. the Taft–Dubois steric parameter *E*_s_’ [36].

The simulation analysis of EPR spectra at both frequencies revealed the following order *g*_‖_(**1**) > *g*_‖_(**2**) > *g*_‖_(**3**) > *g*_z_(**4**) (Table 1), which may reflect the changes in the interaction along the *z*-axis due to the steric effect of the substituent directly bound to the terminal amine nitrogen (Scheme 1). Consequently, the relationship of *g*_‖_ or *g_z_* vs. Taft–Dubois steric parameter E_S_’ was analyzed [36,37]. The linear dependence obtained for complexes **1**–**4** in both frequency domains evidenced the impact of the increased steric hindrance caused by the presence of hydrogen (**1**), methyl (**2**), ethyl (**3**), or phenyl (**4**) groups, on the coordination geometry of the central atom (Figure 2). The EPR spectra of polycrystalline compounds **1**–**3** showed an axial symmetry, while the rhombic spectra were observed for complex **4** which possessed the phenyl substitution with the highest steric demand on the terminal nitrogen (Figure 1, Table 1). 

### 2.2. Cu(II) Complexes in Solution

**Figure 3:**
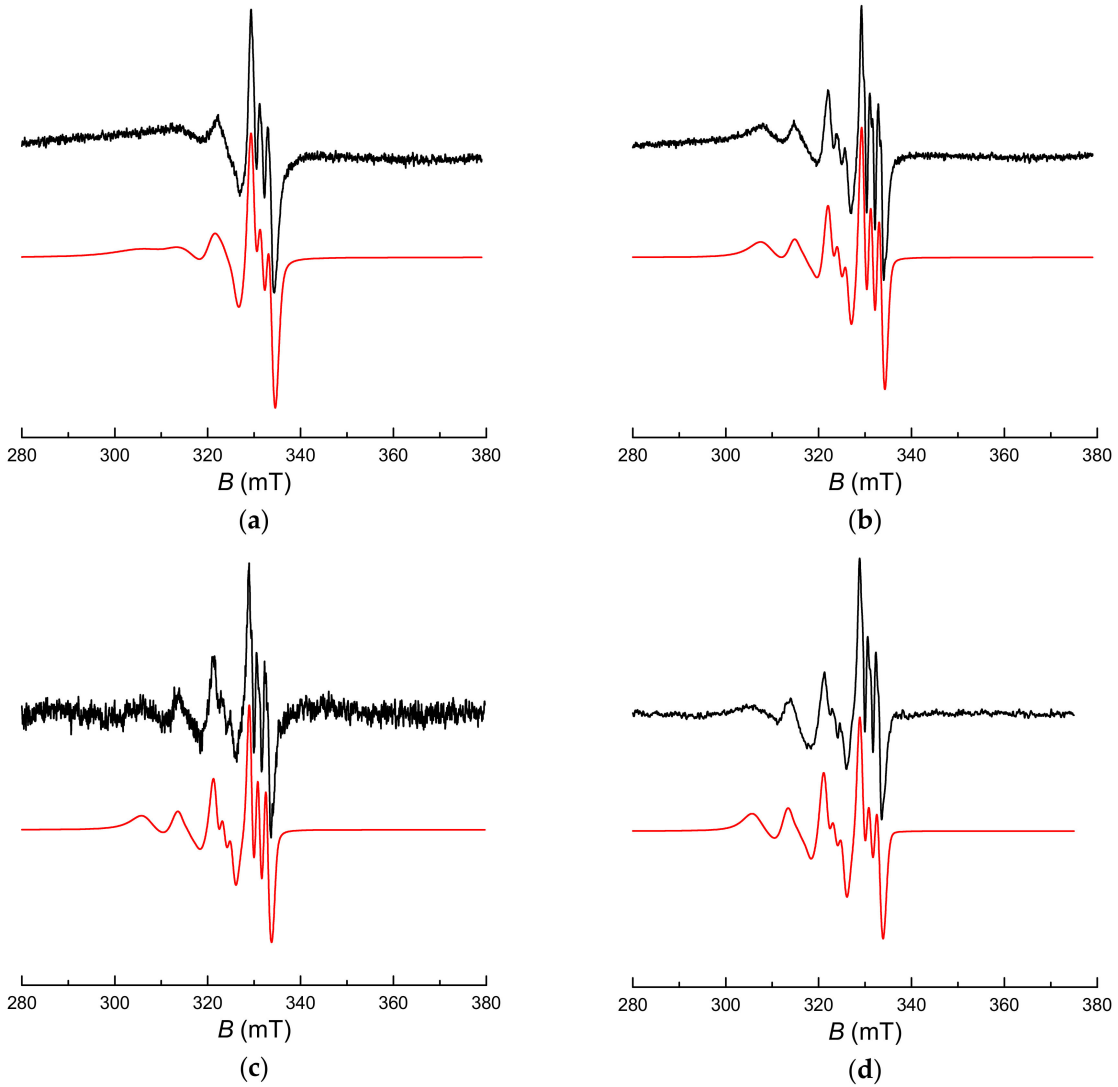
The experimental (**‒**) and simulated (**‒**) EPR spectra of **4** in DMSO (0.8 mM) at 298 K (**a**) or 348 K (**b**) and in H_2_O (0.4 mM) at 298 K (**c**); EPR spectrum obtained by mixing the equimolar (1 mM) aqueous solutions of proligand **L4** (pH = 7) and CuSO_4_ at 298 K, along with its simulation (**d**). EPR spectrometer settings: microwave frequency, 9.424 GHz; microwave power, 10.01 mW; center field, 329.7 mT; gain, 2.0 × 10^5^; modulation amplitude, 0.2 mT; scan, 42 s; time constant, 10.24 ms; number of scans 25. The spin-Hamiltonian parameters elucidated from the simulations are summarized in Table 2.

The Cu(II) complexes **1**–**4**, as well as the corresponding proligands, reveal a moderate solubility in water and DMSO, and consequently further EPR and UV-Vis experiments were performed using these solvents. The EPR spectra of Cu(II) complexes **1**–**4,** measured at 298 K in DMSO or water, showed hyperfine coupling of Cu(II) (*I* = 3/2) and one nitrogen nucleus (*I* = 1), due to the equatorial coordination of one nitrogen atom in the 1:1 copper(II)-to-ligand complexes, as illustrated in Figure 3 for complex **4**. The nitrogen hyperfine coupling was only partially resolved, especially in the more viscous DMSO [37], due to restricted molecular tumbling, leading to line-broadening [38,39,40]. Consequently, in order to improve the resolution, the EPR spectra were measured in the temperature range of 298–348 K. The temperature increase led to a better resolved nitrogen hyperfine splitting without any significant changes of the Cu(II) pattern (Figure 3b). The analogous effect of temperature increase was found in both solvents. The spin-Hamiltonian parameters for **1**–**4 **(*g*, *A*_Cu_ and *A*_N_; summarized in Table 2 for 298 K) were elucidated from the detailed simulations of the X-band EPR spectra. The EPR spectrum of **4** was also measured in a frozen DMSO solution at 100 K, and the averaging of the anisotropic spin-Hamiltonian parameters elucidated from its simulation (*g_x_* = 2.057, *g_y_* = 2.040, *g_z_* = 2.227; *A*_Cu,*x*_ = 2.30 mT, *A*_Cu,*y*_ = 1.78 mT, *A*_Cu,*z*_ = 18.07 mT; *A*_N,*x*_ = 1.46 mT, *A*_N,*y*_ = 1.82 mT, *A*_N,*z*_ = 2.23 mT), gave the values of *g*_av_ = 2.108, *A*_Cu,av_ = 7.38 mT and *A*_N,av_ = 1.84 mT, which matched well with the corresponding parameters obtained from the spectra measured in DMSO solutions at 298 K (Table 2). This confirms that the coordination mode is preserved after freezing, analogously to the previously described Cu(II) complex of salicylaldehyde thiosemicarbazone (STSC) in water [38]. 

Figure 3 shows the EPR spectra of **4** in DMSO at varying temperatures (298 K and 348 K), in water at 298 K, along with the spectra acquired at 298 K by mixing the equimolar (1 mM) aqueous solutions of the proligand **L4** (pH = 7) and CuSO_4_ (Figure 3d). Identical EPR spectra were found for **4** in water and in equimolar mixture **L4**/CuSO_4_ (Figure 3c,d), evidencing the formation of the 1:1 Cu(II)-to-ligand complex via the ONS-donor set in the mixture under given experimental conditions. Analogously, the addition of an equimolar buffered solution (pH = 7) of proligands **L1**–**L3** to CuSO_4_ in water led to the immediate formation of Cu(II) complexes, with the EPR spectra fully compatible with those measured in the aqueous solutions of **1**–**3**. Such behavior of proligands matches well with the results of our previous spectrophotometric investigations of complex formation in the aqueous solutions containing **L1**–**L4** and copper(II) ions [28].

**Figure 4:**
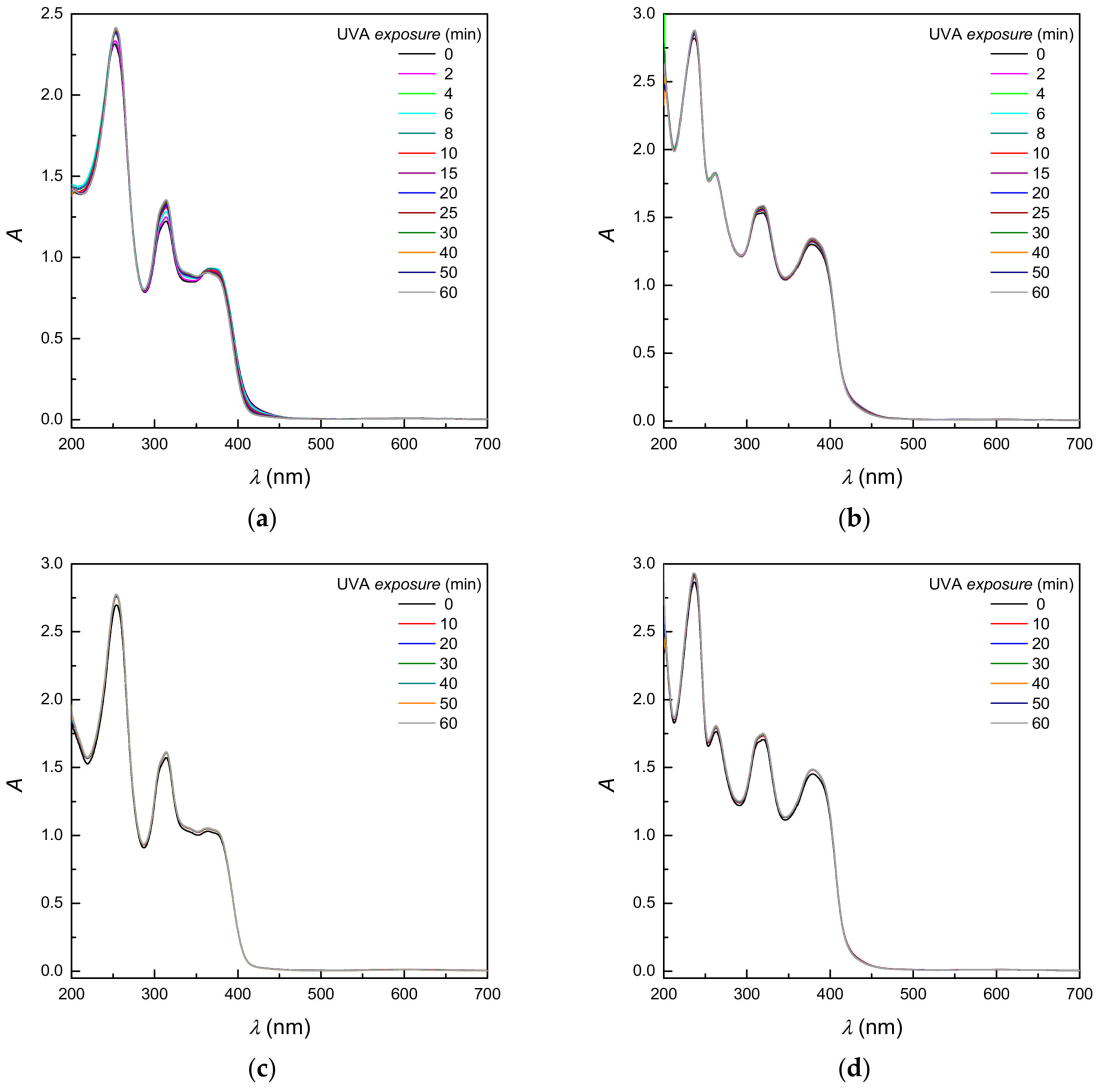
The electronic absorption spectra of the equimolar mixture **L1**/CuSO_4_ (**a**) and **L4**/CuSO_4_ (**b**) in comparison with the spectra of **1** (**c**) and **4** (**d**) in aqueous solutions at 298 K, before and upon discontinuous UVA exposure (*λ*_max_ = 365 nm, irradiance 13 mW cm^−2^; *c*_0_(**L1**,**L4**) = *c*_0_(CuSO_4_) = *c*_0_(**1**,**4**) = 0.1 mM, optical path length 1 cm).

As described above, the EPR spectra obtained by mixing the equimolar aqueous solutions of proligand **L4** and CuSO_4_ are fully compatible with those measured for **4** in aqueous solutions (Figure 3c,d). The formation of 1:1 Cu(II)-to-ligand complexes in aqueous solutions **L1**–**L4**/Cu(II) have been previously observed by UV-Vis spectroscopy [28]. Analogous behavior was confirmed also in our study using buffered (pH = 7) proligand solutions. Figure 4 summarizes the UV-Vis spectra measured in the aqueous solutions of Cu(II) complexes **1** and **4**, along with the electronic spectra found by mixing CuSO_4_ with **L1** or **L4**. All absorption maxima of **L1**/CuSO_4_ and **1** (253 nm, 313 nm and 368 nm) as well as **L4**/CuSO_4_ and **4** (237 nm, 262 nm, 320 nm and 380 nm), along with a broad low-intensity d–d transition maximum around 600 nm, are located at the same positions and possess comparable intensities. This is evidence for the presence of identical 1:1 Cu(II)-to-ligand complexes in aqueous solutions. The addition of Cu(II) ions to the buffered proligand solutions led to the formation of the corresponding 1:1 complex with the characteristic absorption band (*λ*_max_ ~ 375 nm), followed by a negligible intensity decrease in time (60-min period, data not shown), most probably reflecting the equilibrium balance at the given pH as previously described [28]. 

**Figure 5:**
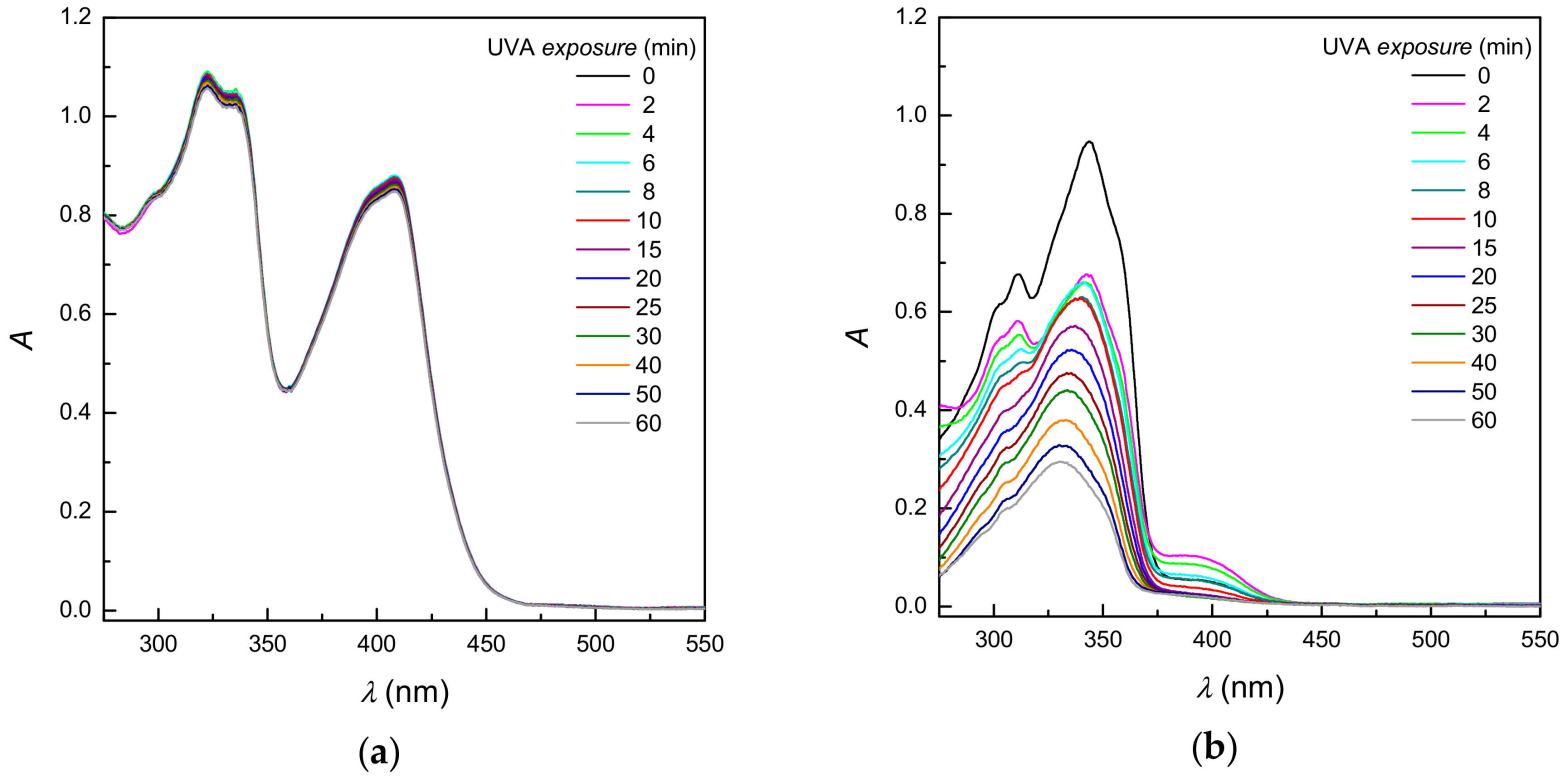
The changes in the electronic absorption spectra monitored upon discontinuous UVA irradiation (*λ*_max_ = 365 nm, irradiance 13 mW cm^−2^) of 0.1 mM DMSO solutions of **4** (**a**) and **L4** (**b**). Optical path length 1 cm, temperature 298 K.

With the aim to investigate the photoinduced generation of reactive oxygen species upon UVA photoexcitation of Cu(II) complexes **1**–**4**, which may improve their cytotoxic action, the changes in the electronic spectra of these complexes upon steady-state exposure, using a monochromatic UVA radiation source (*λ*_max_ = 365 nm), were monitored. Only small variations were found in the electronic spectra during exposure of Cu(II) complexes **1**–**4** dissolved in water, as well as upon irradiation of aqueous solutions prepared by mixing **L1**–**L4**/CuSO_4_ (Figure 4). The analogous photostability of **1**–**4** was confirmed also in DMSO solutions upon UVA photoexcitation as illustrated in Figure 5a for complex **4**, in contrast to a significant photoinduced transformation of the proligand **L4,** which occurred under identical experimental conditions (Figure 5b). Moreover, the results of the EPR spin trapping experiments with DMPO in water, or DMSO, evidenced only very limited generation of ROS upon in situ irradiation of **1**–**4** (data not shown). We suppose that the presence of Cu(II) ions causes the quenching of the excited states of the complexes **1**–**4** [41,42], resulting in a significant decrease of their ability to activate molecular oxygen and produce ROS upon photoexcitation. 

Our previous experiments with Cu(II) complexes **1**–**4** showed a moderate anticancer activity towards human cancer cell lines coupled with a marked ROS accumulation. Hydrogen peroxide is now accepted as a normal metabolite of oxygen in the aerobic metabolism of cells and tissues [43,44], and its interaction with Cu(II) ions can result in the generation of highly reactive and damaging intermediates [45,46]. A simplified redox behavior of the Cu(II) ion in the reaction with hydrogen peroxide is summarized in Equations (1)–(8), considering the existence of copper ions in the oxidation states +1, +2 and +3 [47]:

(1) $$\mathrm{Cu}(\mathrm{II})\;+\;H_{2}O_{2}\overset{k\;=\;{461\;M}^{-1}\;s^{-1}}{\to }\;\mathrm{Cu}(I)\;+\;{2H}^{+}\;+\;O_{2}^{•-}$$

(2) Cu(I) + H2O2 →k = 4.5×105 M-1 s-1 Cu(II) + OH– + O•H

(3) $$\mathrm{Cu}(I)\;+\;H_{2}O_{2}\;\overset{k\;=\;{61\;M}^{-1}\;s^{-1}}{\to }\;\mathrm{Cu}(\mathrm{III})\;+\;{2\mathrm{OH}}^{–}$$

(4) $$\mathrm{Cu}(\mathrm{III})\;+\;H_{2}O_{2}\;\overset{k\;=\;1.3\times 10^{5}{\;M}^{-1}\;s^{-1}}{\underset{}{⇌}}\;\mathrm{Cu}(\mathrm{II})\;+\;{2H}^{+}\;+\;O_{2}^{•-}$$

(5) $$\mathrm{Cu}(I)\;\;+\;\;{2H}^{+}\;+\;O_{2}^{•–}\;\;\overset{k\;=\;2\times 10^{9}{\;M}^{-1}\;s^{-1}}{\to }\;\;\mathrm{Cu}(\mathrm{II})\;\;+\;\;H_{2}O_{2}\;$$

(6) $$\mathrm{Cu}(\mathrm{II})\;+\;O_{2}^{•\text{–}}\;\overset{k\;=\;1{.5\;M}^{-1}\;s^{-1}}{\to }\;\mathrm{Cu}(I)\;+\;O_{2}$$

(7) $$\mathrm{Cu}(I)\;+\;O_{2}\;\overset{k\;=\;6.6\times 10^{8}{\;M}^{-1}\;s^{-1}}{\to }\;\mathrm{Cu}(\mathrm{II})\;+\;O_{2}^{\text{•}–}$$

(8) $$\mathrm{Cu}(I)\;+\;\mathrm{Cu}(\mathrm{III})\;\overset{k\;=\;3.5\times 10^{9}{\;M}^{-1}\;s^{-1}}{\to }\;2\mathrm{Cu}(\mathrm{II})$$

**Figure 6:**
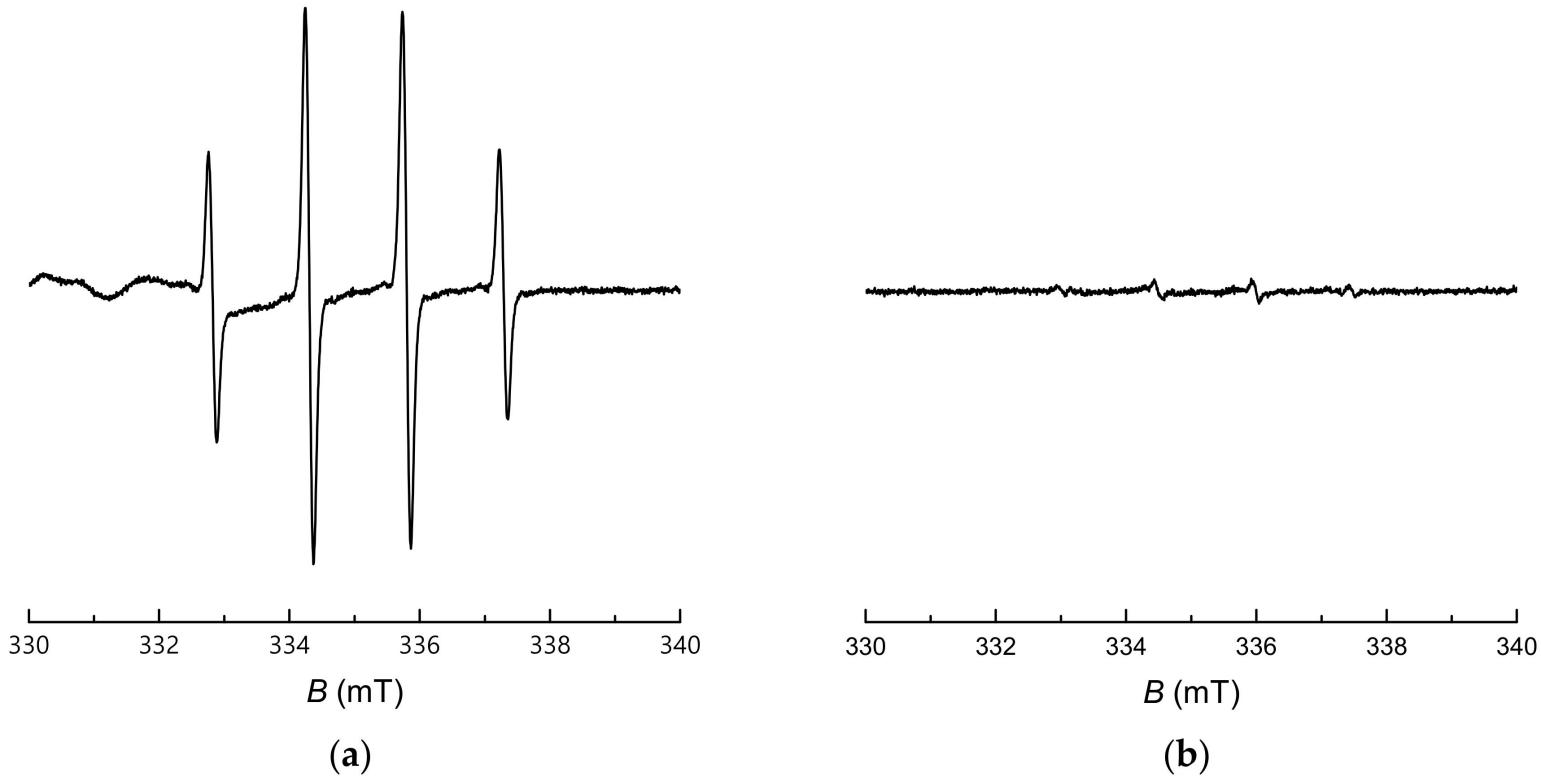
EPR spectrum of the ^•^DMPO-OH spin-adduct measured at 298 K in solutions: (**a**) **2**/H_2_O_2_/DMPO/H_2_O and (**b**) L**2**/H_2_O_2_/DMPO/H_2_O. Initial concentrations: *c*_0_(**2**) = 0.4 mM, *c*_0_(L**2**) = 0.4 mM, *c*_0_(H_2_O_2_) = 0.4 M, *c*_0_(DMPO) = 0.04 M. EPR spectrometer settings: microwave frequency, 9.448 GHz; microwave power, 10.03 mW; center field, 335.0 mT; gain, 2.0 × 10^5^; modulation amplitude, 0.1 mT; scan, 42 s; time constant, 10.24 ms; number of scans 30.

In order to follow the potential interaction of hydrogen peroxide with Cu(II) complexes **1**–**4**, coupled with ROS generation, EPR spin trapping experiments using DMPO spin trap were performed. The addition of hydrogen peroxide to the aqueous solutions of **1**–**4,** containing DMPO, led to the appearance of a four-line EPR signal characterized by the spin-Hamiltonian parameters *A*_N _= 1.485 mT, *A*_H_^β^ = 1.480 mT and *g* = 2.0057, unambiguously assigned to ^•^DMPO-OH spin-adduct [48,49]. Figure 6a depicts the EPR spectrum of ^•^DMPO-OH spin-adduct monitored in an aerated aqueous solution of **2**, in the presence H_2_O_2_ and DMPO. The EPR signal intensity of ^•^DMPO-OH increased proportionally with the increased initial concentration of H_2_O_2_, which was added into the experimental system with fixed concentrations of **1**–**4** complexes. This increase in signal intensity evidences a complicated reaction mechanism of hydroxyl radical generation (Equations (1)–(8)). The exact mechanisms of the reactions proceeding in our system cannot be determined, but the EPR experiments with H_2_O_2_ confirmed the formation of the hydroxyl radical spin-adduct only in the solutions of Cu(II) complexes **1**–**4**. A low-intensity signal of ^•^DMPO-OH was monitored in the solutions of proligands** L1**–**L4** containing H_2_O_2_ and DMPO (Figure 6b), and was also found in the reference system (i.e., in the aqueous DMPO solution containing H_2_O_2_); this reflects the interaction of the reactive spin trap with H_2_O_2_ present at high concentration. The addition of hydrogen peroxide into the aqueous solutions of **1**–**4** had no effect on the intensity of the EPR signal of paramagnetic Cu(II) ions, which correlated with rapid transformation of diamagnetic Cu(I)/Cu(III) to Cu(II) in the aerated H_2_O_2_ solutions [47].

### 2.3. Photoinduced Processes of Proligands Monitored by the EPR Spin Trapping Technique

Many pharmaceutically utilized heterocyclic compounds are known to be sensitive towards UVA exposure, which could either increase or inhibit their biological activity. Such photoinduced processes are often related to the generation of reactive oxygen species via the activation of molecular oxygen by the excited states of the photoactive molecule or directly by the molecular changes in the compound itself [50,51]. Previously, we studied in detail the photoactivation of molecular oxygen upon excitation of different quinolone derivatives (fluoroquinolones, nitroquinolones, selenadiazoloquinolones), and considerable photobiological effects were found for several derivatives [52,53,54,55]. 

**Figure 7:**
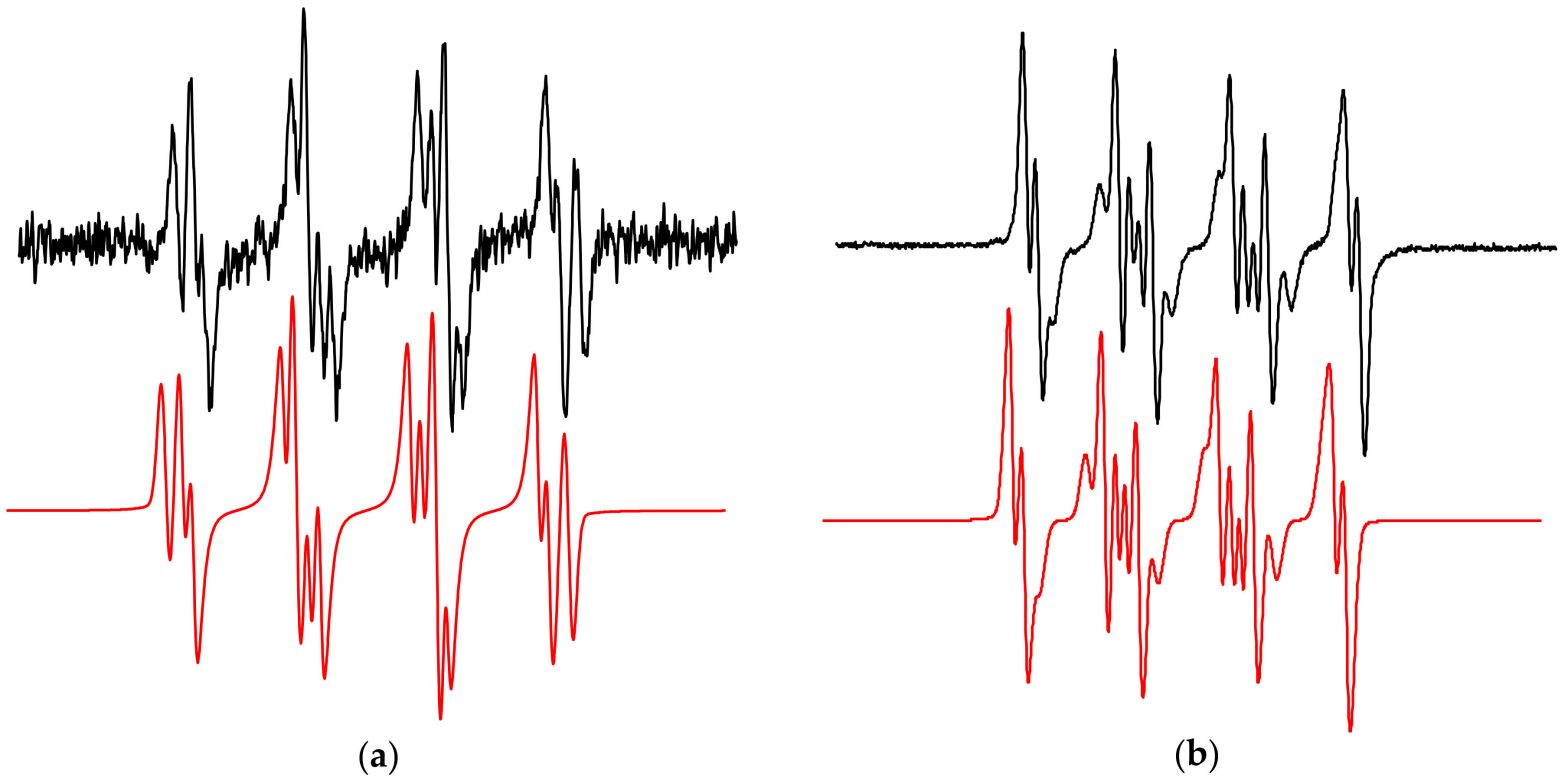
The experimental (**‒**) and simulated (**‒**) EPR spectra monitored upon in situ UVA exposure of proligand **L4** in the buffered (pH = 7) (**a**) or DMSO (**b**) solution, in the presence of a DMPO spin trapping agent. (*c*_0_(**L4**) = 0.8 mM, *c*_0_(DMPO) = 0.04 M, *λ*_max_ = 365 nm, irradiance 13 mW cm^−2^). EPR spectrometer settings: microwave frequency, 9.458 or 9.434 GHz; microwave power, 20.02 or 10.01 mW; center field, 335.2 mT; magnetic field sweep, 8 mT; gain, 5.02 × 10^5^ or 2.0 × 10^5^; modulation amplitude, 0.1 or 0.05 mT; scan, 42 s; time constant, 20.48 or 10.24 ms; number of scans 25.

As information regarding the behavior of aromatic thiosemicarbazones upon irradiation is limited [56,57], the ability of proligands **L1**–**L4** to activate oxygen and generate ROS upon their UVA irradiation was investigated in aerated aqueous (pH = 7) or DMSO solutions, using EPR spin trapping with DMPO. Figure 7 shows the experimental and simulated EPR spectra of DMPO spin-adducts obtained upon the UVA exposure of proligand **L4** in both solvents. The EPR spectrum measured upon irradiation of the system **L4**/DMPO/buffer/air consists of two signals. The dominant twelve-line signal of ^•^DMPO-OOH with spin-Hamiltonian parameters *A*_N _= 1.417 mT, *A*_H_^β^ = 1.197 mT, *A*_H_^γ ^= 0.118 mT; *g* = 2.0058 [48], is superimposed on the four-line signal typical of a ^•^DMPO-OH spin-adduct. However, in aqueous systems, the photogenerated O_2_^•−^/^•^OOH is readily transformed into H_2_O_2_ [58], which can be involved in the consecutive (photo)reactions producing ^•^OH [49]. After the radiation was switched off, the signal of ^•^DMPO-OOH was transformed to ^•^DMPO-OH, reflecting the well-known behavior and low stability of ^•^DMPO-OOH in aqueous media [59]. The analogous EPR spectra of DMPO spin-adducts were obtained upon UVA exposure of all proligands **L1**–**L4** in buffer, thus the formation of the ^•^DMPO-OOH spin-adduct evidenced the interaction of their photoexcited molecules with molecular oxygen via the electron transfer mechanism [60].

The increased stability of the photogenerated superoxide radical anion in aprotic media [58] is well documented by the enhanced intensity of the corresponding spin-adduct EPR signal, measured upon irradiation of solution **L4**/DMPO/DMSO/air (Figure 7b). The in situ photoactivation of **L4** in the aerated DMPO/DMSO solution resulted in the immediate generation of a characteristic twelve-line spectrum with spin-Hamiltonian parameters (*A*_N _= 1.275 mT, *A*_H_^β^ = 1.033 mT, *A*_H_^γ ^= 0.138 mT; *g* = 2.0057), which was fully compatible with the ^•^DMPO-O_2_^−^ spin-adduct [48] found as a major component of the solution (relative concentration 66%). Simulation analysis of the experimental spectrum shown in Figure 7b revealed the superoxide radical anion as well as the presence of other paramagnetic species, generated via interaction with DMSO [49,61], trapped as a ^•^DMPO-OCH_3_ spin-adduct (*A*_N _= 1.314 mT, *A*_H_^β^ = 0.800 mT, *A*_H_^γ ^= 0.167 mT; *g* = 2.0057, relative concentration 34%). The generation of the ^•^DMPO-O_2_^−^ spin-adduct monitored upon UVA excitation of **L1**–**L4** in DMSO solutions confirmed the activation of molecular oxygen, via photoexcitated proligand molecules. As mentioned above, the addition of an equimolar concentration of Cu(II) ions to a proligand solution or dissolved Cu(II) complexes **1**–**4** did not yield any DMPO spin-adducts upon exposure and their photoinduced processes were fully inhibited. 

### 2.4. UVA Photoexcitation of Proligands Monitored by UV-Vis Spectroscopy—Steady-State Experiments

Thiosemicarbazones represent polyfunctional compounds with the potential to cyclize upon UV irradiation; the reaction depends on conditions used as well as the structure of the precursors [56,57]. Previous investigations evidenced that the prolonged UV irradiation of 2-methyl-4-phenyl-substituted benzaldehyde thiosemicarbazones yields the corresponding ∆^2^-1,2,4-triazoline-5-thione derivatives through the formation of stable 1,2,4-triazolidine-5-thione intermediates [57].

**Figure 8:**
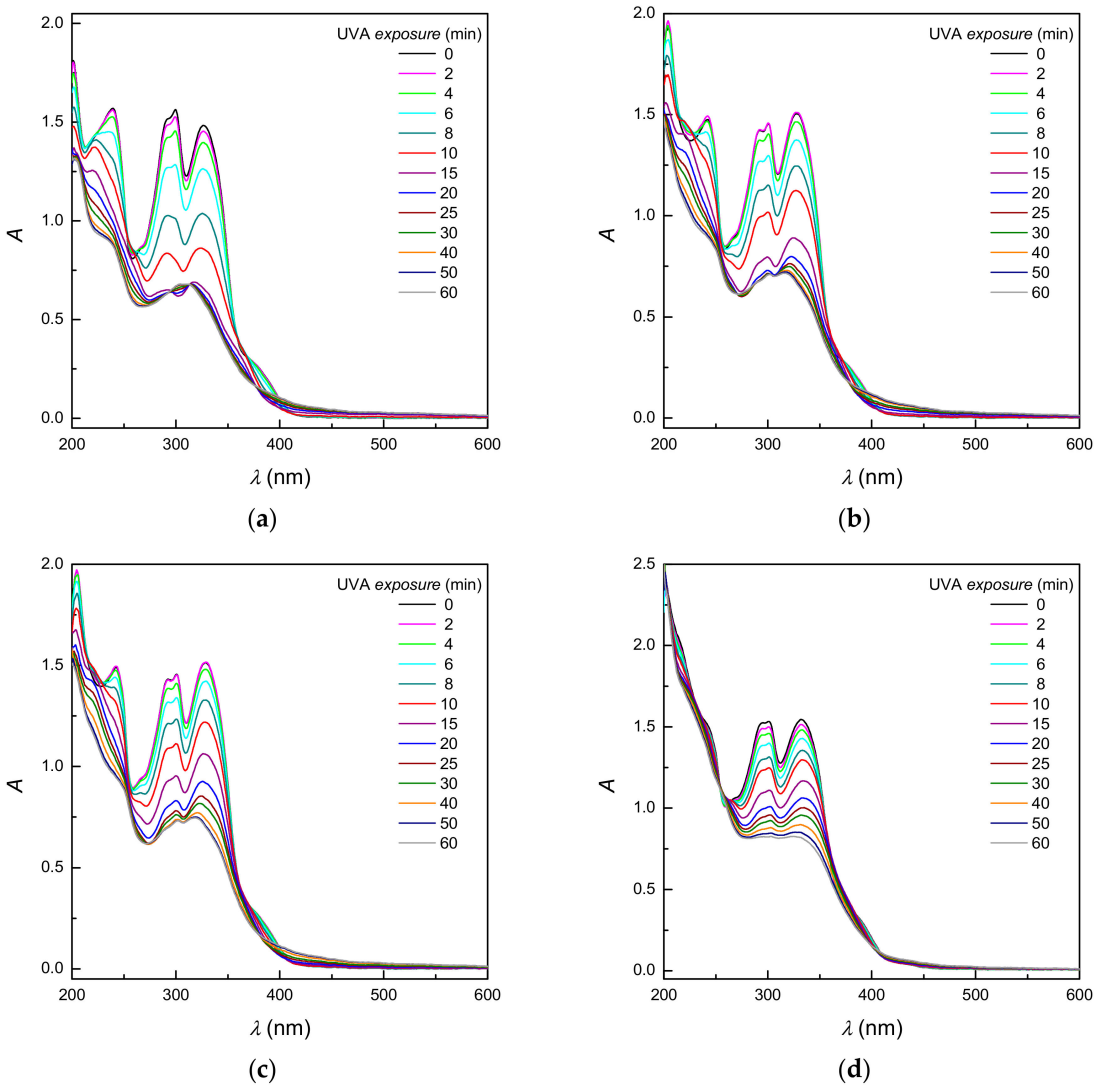
The changes in the electronic absorption spectra monitored upon discontinuous UVA irradiation (*λ*_max_ = 365 nm, irradiance 13 mW cm^−2^) investigating 0.1 mM buffer solutions (pH = 7) of proligand **L1** (**a**), **L2** (**b**), **L3** (**c**) and **L4** (**d**). Optical path length 1 cm, temperature 298 K.

**Figure 9:**
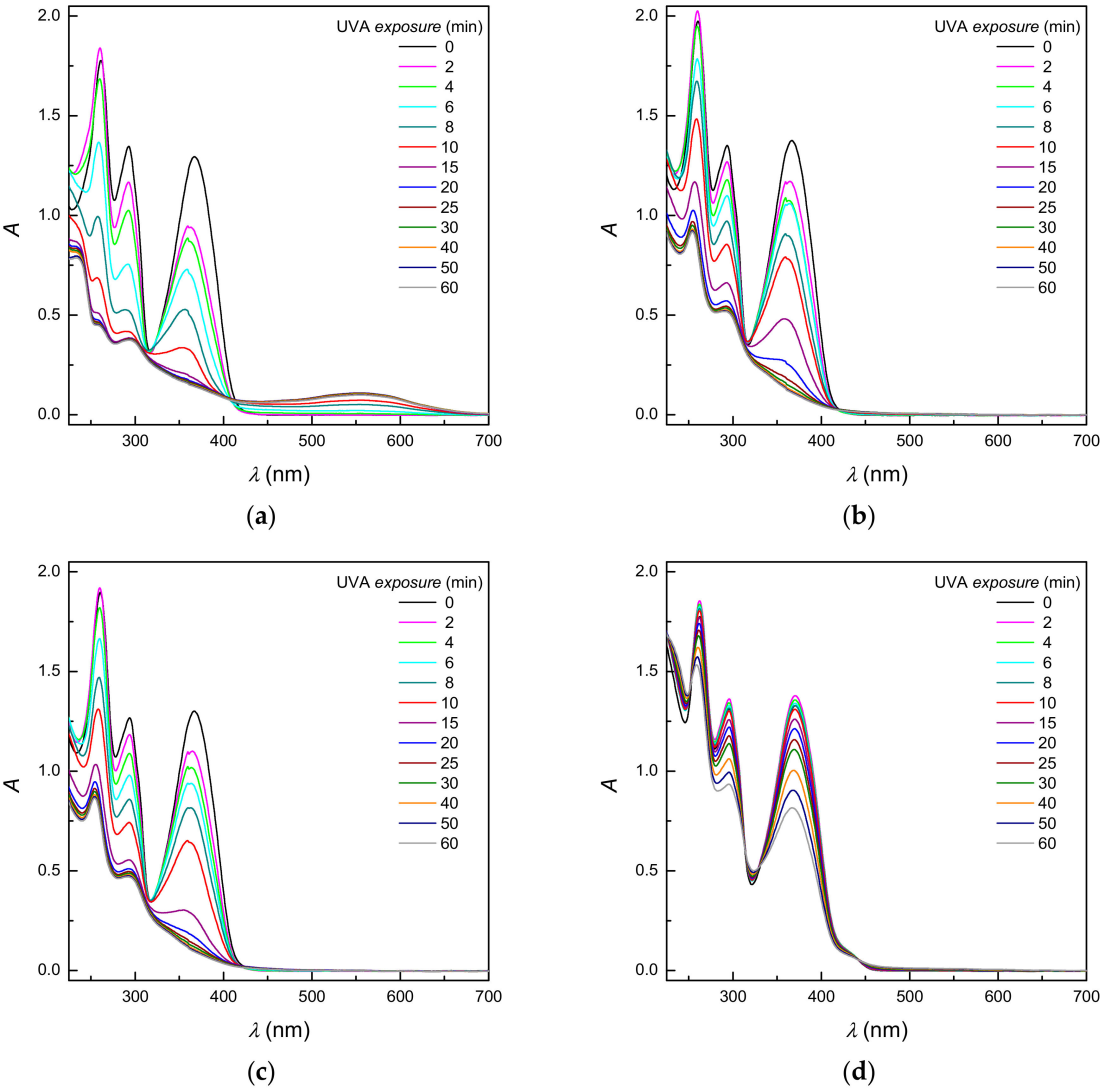
The changes in the electronic absorption spectra monitored upon discontinuous UVA irradiation (*λ*_max_ = 365 nm, irradiance 13 mW cm^−2^) investigating 0.1 mM buffer solutions (pH = 10) of proligand **L1** (**a**), **L2** (**b**), **L3** (**c**) and **L4** (**d**). Optical path length 1 cm, temperature 298 K.

The proton dissociation of proligands **L1**–**L4** in their respective aqueous solutions at different pH values is associated with the characteristic spectral changes in their electronic absorption spectra [28]. The aqueous solutions of **L1**–**L4** are colorless at neutral pH, with the absorption maxima at 240 nm, 300 nm and 327 nm (Figure 8), whereas the absorption maxima were at 260 nm, 292 nm and 367 nm in the alkaline solutions (pH = 10) (Figure 9). Proligands **L1**–**L4** showed a limited photostability, as upon UVA irradiation in the buffered solutions the significant changes were monitored in their electronic absorption spectra, which were only slightly dependent on the substituent character at the terminal nitrogen (Figure 8 and Figure 9). The UVA photoexcitation of proligands **L1**–**L4** at neutral pH resulted in a substantial absorbance decrease at 300 and 327 nm within 60 min. This is typical for aldimine and phenolic chromophores [28] (Figure 8), reflecting their phototransformation. As the EPR spin trapping experiments confirmed the photogeneration of O_2_^•−^/^•^OOH and hydroxyl radicals under analogous experimental conditions, we can suppose that the observed damage of the aldimine and phenolic chromophores was induced by the radical attack via photogenerated ROS. 

The intensive absorption band at 370 nm was found in the electronic spectra in alkaline solutions (pH = 10) due to the deprotonation of the phenolic group of proligands **L1**–**L4**, reflecting the presence of an extended π-conjugated system [28]. Photoexcitation of proligand molecules **L1**–**L4** with UVA radiation (*λ*_max_ = 365 nm) resulted in a substantial decrease in the absorption bands at 250 nm, 300 nm, and 370 nm under aforementioned experimental conditions. The decrease was also associated with the formation of a low-intensity absorption band at ~550 nm, visible especially for proligand **L1** with hydrogen at the terminal nitrogen (Figure 9). The limited photostability of all proligands upon UVA exposure was also monitored in aprotic DMSO, revealing the decline in the absorption band at 345 nm as is shown for **L4** in Figure 5b.

The results of EPR spin trapping experiments and the changes monitored in the electronic absorption spectra of proligands **L1**–**L4** upon UVA exposure confirmed the photoinduced generation of ROS via photoactivation of molecular oxygen and a limited photostability of the proligands in aqueous and DMSO solutions.

## 3. Materials and Methods 

All reagents were used as purchased from commercial suppliers, mainly Sigma Aldrich (Buchs, Switzerland). The 5-sulfonate-salicylaldehyde sodium salts (NaH_2_L^X^; X = H, Me, Et, Ph; **L1**–**L4** in Scheme 1) and their Cu(II)-complexes (Cu^II^HL^X^(Y); X = H, Me, Et, Ph; Y = DMSO, H_2_O; **1**–**4** in Scheme 1) were prepared by previously described procedures [28]. The spin trapping agent 5,5-dimethyl-1-pyrroline *N*-oxide (DMPO; Sigma-Aldrich, Buchs, Switzerland) was distilled prior to the application. Hydrogen peroxide (≥30%, for trace analysis) was purchased from Sigma-Aldrich and used without further purification. 

The solid-state EPR experiments at room temperature and at 100 K were performed by an EMX Plus EPR spectrometer (Bruker, Rheinstetten, Germany) operating in Q-band in the standard ER 5106 QT Q-band probe for cw-EPR, and by an EMX EPR spectrometer (Bruker, Rheinstetten, Germany), operating in X-band at 100 kHz field modulation in the standard TE_102_ (ER 4102 ST) rectangular cavity. Thin-walled quartz EPR tubes (Bruker) were used in all solid-state experiments. The temperature was lowered to 100 K by the temperature control unit ER 4141VT-U (Q-band; Bruker) or ER 4111 VT (X-band; Bruker), with liquid nitrogen. The *g*-values were determined by the simultaneous measurement of a reference sample containing Mn(II)/MgO standard, a standard strong pitch (Bruker) placed on the wall of the EPR cell or using a nuclear magnetic resonance teslameter (ER 036TM, Bruker, Rheinstetten, Germany) and integrated frequency counter. The effect of temperature on the EPR spectra of Cu(II) complexes **1**–**4** in the aqueous or DMSO solution was monitored over the temperature range 298–348 K; a Bruker temperature control unit ER 4111 VT was used to adjust the temperature. The experimental EPR spectra were processed and analyzed by the Bruker software WinEPR, and the simulated spectra were calculated using the SimFonia program (Bruker) or EasySpin 5.1.10 simulation toolbox [62] working under Matlab (MathWorks, Natick, MA, USA) software.

The generation of paramagnetic intermediates was monitored by cw-EPR spectroscopy using the EMX spectrometer. Freshly prepared 1 mM solutions of Cu(II) complexes **1**–**4** or proligands **L1**–**L4** were mixed with the solution of spin trapping agent DMPO before the EPR measurements, carefully aerated and immediately transferred to a flat quartz cell (WG 808-Q, Wilmad-LabGlass, Vineland, NJ, USA). The samples were irradiated at 298 K directly in ER 4102 ST cavity and the EPR spectra were measured in situ. The UV LED monochromatic radiator (*λ*_max_ = 365 nm, irradiance 13 mW cm^−2^; Bluepoint LED, Hönle UV Technology, Gräfelfing/München, Germany) was used as the source of monochromatic UVA light. The experimental EPR spectra of spin-adducts were simulated using Winsim2002 software (NIEHS, Durham, NC, USA) suitable for analysis and fitting of multi-component isotropic EPR spectra of nitroxide radicals [63].

The UV-Vis spectra of thiosemicarbazone Cu(II) complexes **1**–**4** and proligands **L1**–**L4** were measured on a UV-3600 UV/vis/NIR (Shimadzu, Kyoto, Japan) spectrophotometer at 298 K. A cell holder (TCC-240A, Shimadzu) was used to control the temperature. The sets of steady-state experiments were performed to monitor the changes in the electronic absorption spectra upon discontinuous irradiation in aqueous or DMSO solutions. The stock solutions (1 mM) were prepared directly before measurements in deionized water, buffer solutions or dried DMSO (SeccoSolv, Merck, Darmstadt, Germany) and diluted to the final concentration of 0.1 mM. As-prepared solutions were irradiated in a 1-cm rectangular quartz cell (Agilent Technologies, Santa Clara, CA, USA) using a monochromatic LED source (*λ*_max_ = 365 nm, irradiance 13 mW cm^−2^; Bluepoint LED) under the atmospheric conditions. The first spectrum was measured without irradiation; the following spectra were recorded immediately after a defined exposure, until the total irradiation time of 60 min was reached. The spectra acquisition was performed using UV Probe (Shimadzu, Kyoto, Japan) software and processed by OriginPro (OriginLab, Northampton, MA, USA) program. The pH values of solutions were measured by a Jenway 3520 pH Meter (Jenway, Stone, Staffordshire, UK) using a glass combination pH electrode (Sentek P13, Essex, UK); Jenway pH buffers (pH 4, 7 and 9) were used for calibration.

## 4. Conclusions

EPR spin trapping experiments showed that, upon UVA exposure, the proligands **L1**–**L4** behave as photosensitizers generating the superoxide radical anion and hydroxyl radical via photoactivation of molecular oxygen. Addition of an equimolar amount of Cu(II) ions into the aqueous proligand solutions resulted in 1:1 complex formation through the ONS-donor set, with the EPR and electronic absorption spectra fully compatible with those found for the solutions of corresponding Cu(II) complexes **1**–**4**. The photoactivation of molecular oxygen and ROS generation is significantly hindered upon UVA photoexcitation of Cu(II) complexes. However, the dark redox activity of the Cu(II) central ion with hydrogen peroxide resulted in the formation of hydroxyl radicals, detected as the corresponding ^•^DMPO-OH spin-adduct.

## Figures and Tables

**Table 1:** Spin-Hamiltonian parameters elucidated from the simulations of the experimental EPR spectra of polycrystalline copper(II) complexes **1**–**4** measured at 100 K.

Cu(II) Complex	Spin-Hamiltonian Parameters							
	X-Band				Q-Band			
	*g*_⊥_^1^		*g*_‖_^1^	*g*_av_^2^	*g*_⊥_^3^		*g*_‖_^3^	*g*_av_^2^
**1**	2.055		2.222	2.111	2.046		2.215	2.102
**2**	2.048		2.199	2.098	2.043		2.212	2.099
**3**	2.067		2.195	2.109	2.058		2.192	2.103
	*g_x_* ^1^	*g_y_* ^1^	*g_z_* ^1^	*g*_av_^2^	*g_x_* ^3^	*g_y_* ^3^	*g_z_* ^3^	*g*_av_^2^
**4**	2.045	2.104	2.132	2.093	2.038	2.099	2.135	2.090

^1^ ±0.002; ^2^ *g*_av_ = (2*g* + *g*_‖_)/3 for axial, *g*_av_ = (*g_x_* + *g_y_* + *g_z_*)/3 for rhombic symmetry; ^3^ ±0.003.

**Table 2:** Spin-Hamiltonian parameters elucidated from the simulations of the experimental X-band EPR spectra of copper(II) complexes **1**–**4** measured at 298 K in DMSO and water.

Cu(II) Complex	*g*-Value^ 1^				Hyperfine Coupling (mT)							
	*g_x_*	*g_y_*	*g_z_*	*g*_av_	*A*_Cu,*x*_	*A*_Cu,*y*_	*A*_Cu,*z*_	*A*_Cu,av_	*A*_N,*x*_	*A*_N,*y*_	*A*_N,*z*_	*A*_N,av_
	*Dimethylsulfoxide*											
**1**	2.0994	2.0935	2.0944	2.0958	7.02	7.44	6.92	7.13	2.16	1.46	1.73	1.78
**2**	2.0923	2.0850	2.0934	2.0902	6.99	7.43	7.21	7.21	2.05	1.61	1.81	1.82
**3**	2.0909	2.0938	2.0945	2.0931	7.02	7.39	7.38	7.26	1.87	1.71	1.67	1.75
**4**	2.0966	2.0931	2.1073	2.0990	7.13	7.46	7.59	7.39	1.75	1.81	1.88	1.81
	*Water*											
**1**	2.0955	2.1049	2.0997	2.1000	6.89	7.30	7.38	7.19	1.97	1.76	1.69	1.81
**2**	2.0931	2.1007	2.0965	2.0968	7.37	7.28	7.53	7.39	1.93	1.66	1.56	1.72
**3**	2.0967	2.0989	2.0968	2.0975	7.28	7.38	7.23	7.30	1.70	1.73	1.81	1.75
**4**	2.0971	2.0948	2.1065	2.0995	7.20	7.15	7.93	7.43	1.93	1.59	1.63	1.72

^1^ ±0.0005.
